# Internal auditory canal volume in normal and malformed inner ears

**DOI:** 10.1007/s00405-022-07676-1

**Published:** 2022-10-09

**Authors:** David Bächinger, Tabita M. Breitsprecher, Alexander Pscheidl, Anandhan Dhanasingh, Robert Mlynski, Stefan Dazert, Sönke Langner, Nora M. Weiss

**Affiliations:** 1 Department of Otorhinolaryngology-Head and Neck Surgery, Ruhr-University Bochum, St. Elisabeth-Hospital Bochum, Bochum, Germany; 2 Department of Otorhinolaryngology, Head and Neck Surgery, University Hospital Zurich, Zurich, Switzerland; 3 University of Zurich, Zurich, Switzerland; 4 Department of Otorhinolaryngology, Head and Neck Surgery, Medical Center, Dortmund, Germany; 5 MED-EL, Innsbruck, Austria; 6 Department of Translational Neurosciences, Faculty of Medicine and Health Sciences, University of Antwerp, Antwerp, Belgium; 7 Department of Otorhinolaryngology, Head and Neck Surgery, “Otto Körner”, Rostock University Medical Center, Rostock, Germany; 8 Institute of Diagnostic and Interventional Radiology, Pediatric and Neuroradiology, Rostock University Medical Center, Rostock, Germany; 9 International Graduate School of Neuroscience (IGSN), Ruhr-University Bochum, Bochum, Germany

**Keywords:** Internal auditory canal, Cochlear malformation, Inner ear malformation, Diagnosis, Volume, 3D segmentation

## Abstract

**Purpose:**

A narrow bony internal auditory canal (IAC) may be associated with a hypoplastic cochlear nerve and poorer hearing performances after cochlear implantation. However, definitions for a narrow IAC vary widely and commonly, qualitative grading or two-dimensional measures are used to characterize a narrow IAC. We aimed to refine the definition of a narrow IAC by determining IAC volume in both control patients and patients with inner ear malformations (IEMs).

**Methods:**

In this multicentric study, we included high-resolution CT (HRCT) scans of 128 temporal bones (85 with IEMs: cochlear aplasia, *n* = 11; common cavity, *n* = 2; cochlear hypoplasia type, *n* = 19; incomplete partition type I/III, *n* = 8/8; Mondini malformation, *n* = 16; enlarged vestibular aqueduct syndrome, *n* = 19; 45 controls). The IAC diameter was measured in the axial plane and the IAC volume was measured by semi-automatic segmentation and three-dimensional reconstruction.

**Results:**

In controls, the mean IAC diameter was 5.5 mm (SD 1.1 mm) and the mean IAC volume was 175.3 mm^3^ (SD 52.6 mm^3^). Statistically significant differences in IAC volumes were found in cochlear aplasia (68.3 mm^3^, *p* < 0.0001), IPI (107.4 mm^3^, *p* = 0.04), and IPIII (277.5 mm^3^, *p* = 0.0004 mm^3^). Inter-rater reliability was higher in IAC volume than in IAC diameter (intraclass correlation coefficient 0.92 vs. 0.77).

**Conclusions:**

Volumetric measurement of IAC in cases of IEMs reduces measurement variability and may add to classifying IEMs. Since a hypoplastic IAC can be associated with a hypoplastic cochlear nerve and sensorineural hearing loss, radiologic assessment of the IAC is crucial in patients with severe sensorineural hearing loss undergoing cochlear implantation.

## Introduction

Cochlear implant (CI) surgery is the current gold-standard treatment in profound sensorineural hearing loss. Among other factors, a narrow bony internal auditory canal (IAC) may be associated with poorer hearing performances after cochlear implantation as a narrow IAC commonly contains a hypoplastic cochlear nerve [1, 2, 3, 4, 5]. Furthermore, IAC hypoplasia may be associated with an aberrant facial nerve, which renders the surgical approach to the cochlea more challenging and the risk for intraoperative facial nerve injury is increased [1, 6].

A narrow IAC can lead to an inferior CI outcome due to various reasons. First, a narrow IAC may contain a rudimentary or absent cochlear nerve [7]. While cochlear nerve aplasia is a contraindication to cochlear implantation [8, 9], cochlear nerve hypoplasia is associated with worse hearing outcomes in CI patients [3, 4, 5]. Moreover, previous studies have reported that a narrow IAC can be associated with both a normal inner ear as well as inner ear malformations (IEMs) [5, 10]. While it has been previously suggested that development of a normal cochlea depends on the innervation by a normally developed cochlear nerve, recent temporal bone studies revealed that the cochlea undergoes a normal development even in absence of the cochlear nerve [11, 12]. However, among candidates for CI surgery, patients with IEMs not only pose significant anatomical challenges to CI surgery, but also have been shown to have a worse outcome regarding hearing performance [5, 13, 14]. Yet, the relation between different types of IEMs, IAC hypoplasia and cochlear nerve abnormalities are not well-understood.

The first definition of a normal IAC was presented in 1964 and together with subsequent studies, a diameter less than 2 mm has become the most widely accepted criterion for a narrow IAC [2, 15, 16, 17]. Currently, there is no broad consensus on how to assess and grade the IAC size. In our previous work, we found that three-dimensional reconstruction and volumetric measurements of temporal bone structures reduce inter-observer differences compared to two-dimensional measurements and may, therefore, help to distinguish normal from abnormal anatomic structures, such as the cochlea or the vestibular aqueduct [18, 19, 20, 21]. Therefore, we hypothesized that three-dimensional volumetric measurements may be a valuable tool to determine the IAC size. A narrow bony IAC can indicate a hypoplastic auditory nerve and thus poorer hearing outcome. Therefore, the assessment of the IAC size may help to identify hypoplasia of the auditory nerve in IEM. In this study, we aimed to (i) estimate metric and volumetric reference values for the IAC of normal and malformed inner ear, (ii) assess the relationship between IAC diameter and volume, and (iii) report the incidence of IAC hypoplasia in different types of IEM.

## Methods

In a retrospective multi-center study, high-resolution CT (HRCT) data sets from patients with IEM and controls were analyzed. Data sets were anonymized prior to image analyses. The study protocol was approved by the local ethics committee (No. 21-7358-BR).

### Image analysis

**Fig. 1:**
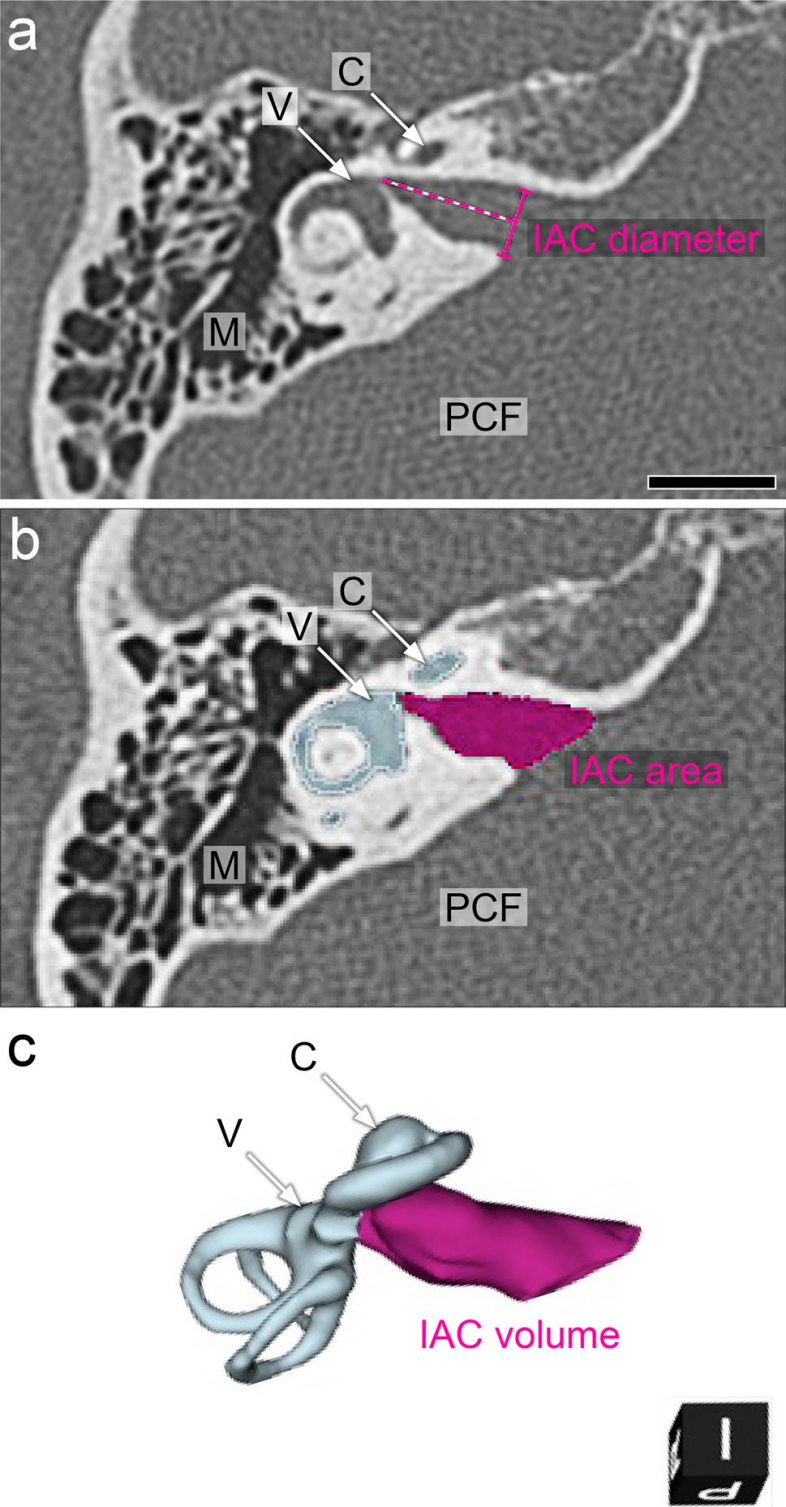
Exemplary measurements of the internal auditory canal (IAC) diameter and volume of a normal inner ear in an axial section of a high-resolution computed tomography. **A** At the *porus acusticus internus*, the IAC diameter (magenta line) was determined perpendicular to the longitudinal axis (dashed magenta line) of the IAC. Scale bar: 10 mm. **B** Exemplary segmentation of the IAC (magenta area). The cochlea (C) and vestibule (V) are outlined in light blue. **C** 3D reconstruction of the IAC (magenta) and the adjacent inner ear (cochlea, vestibule; light blue) M, mastoid. PCF, posterior cranial fossa

The IAC diameter was assessed from HRCT data sets in the axial view. IAC diameter was determined at the *porus acusticus internus* (Fig. 1A) [22]. HRCT data sets were reconstructed using 3D slicer (https://www.slicer.org/, version 4.13.0, Massachusetts, USA [23]). Segmentation of the inner ear and IAC was performed using threshold analysis (threshold range: − 1024 to 700 Hounsfield units, Fig. 1B) and a three-dimensional model of the inner ear was reconstructed as previously described (Fig. 1C) [24]. The IAC was segmented from its fundus to the posterior cranial fossa. Volumes were calculated using the segment statistics module and the segmentation module of the 3D slicer software.

IEMs were diagnosed according to the Sennaroglu and Saatci classification [25, 26] by a senior neuroradiologist with more than 10 years of experience in temporal bone radiology. All measurements were performed by two independent  examiners with at least 2 years of experience in the diagnosis of temporal bone imaging and after an intensive training period by a senior otorhinolaryngologist and a senior neuroradiologist.

### Statistical analysis

Statistical analyses were performed using Prism (version 8, GraphPad Software, La Jolla, CA, USA). Values given as mean and standard deviation (SD). To compare differences among group means, a one-way analysis of variance (ANOVA) was used. Groups with a number *n* < 3 were excluded from statistical analysis. Tukey’s test was used to correct for multiple comparisons. The significance level was set to *p* < 0.05. Correlations were assessed using Pearson’s correlation. A Person’s correlation coefficient of < 0.3 was interpreted as an indicator of a weak correlation, 0.3–0.59 of a fair correlation, 0.6–0.79 of a moderate correlation, and 0.8–0.99 of a very strong correlation [27, 28]. The inter-rater reliability (IRR) was determined by calculating the intra-class correlation coefficient (ICC).

## Results

### Demographics

HRCT of the temporal bone of 83 IEM patients and 45 controls with no inner ear pathology were analyzed. Slice thickness varied between 0.625 and 1 mm. The group of IEMs consisted of 11 cases of cochlear aplasia (CA), 2 cases of common cavity (CC), 3 cases of cochlear hypoplasia type I, 2 cases of cochlear hypoplasia type III, 14 cases of cochlear hypoplasia type IV, 8 cases of incomplete partition (IP) type I, 8 cases of IP type III, 16 cases of IP type II with an enlarged vestibular aqueduct (Mondini malformation [MM]) and 19 cases of enlarged vestibular aqueduct syndrome (EVAS).

### Internal auditory canal diameter

**Fig. 2:**
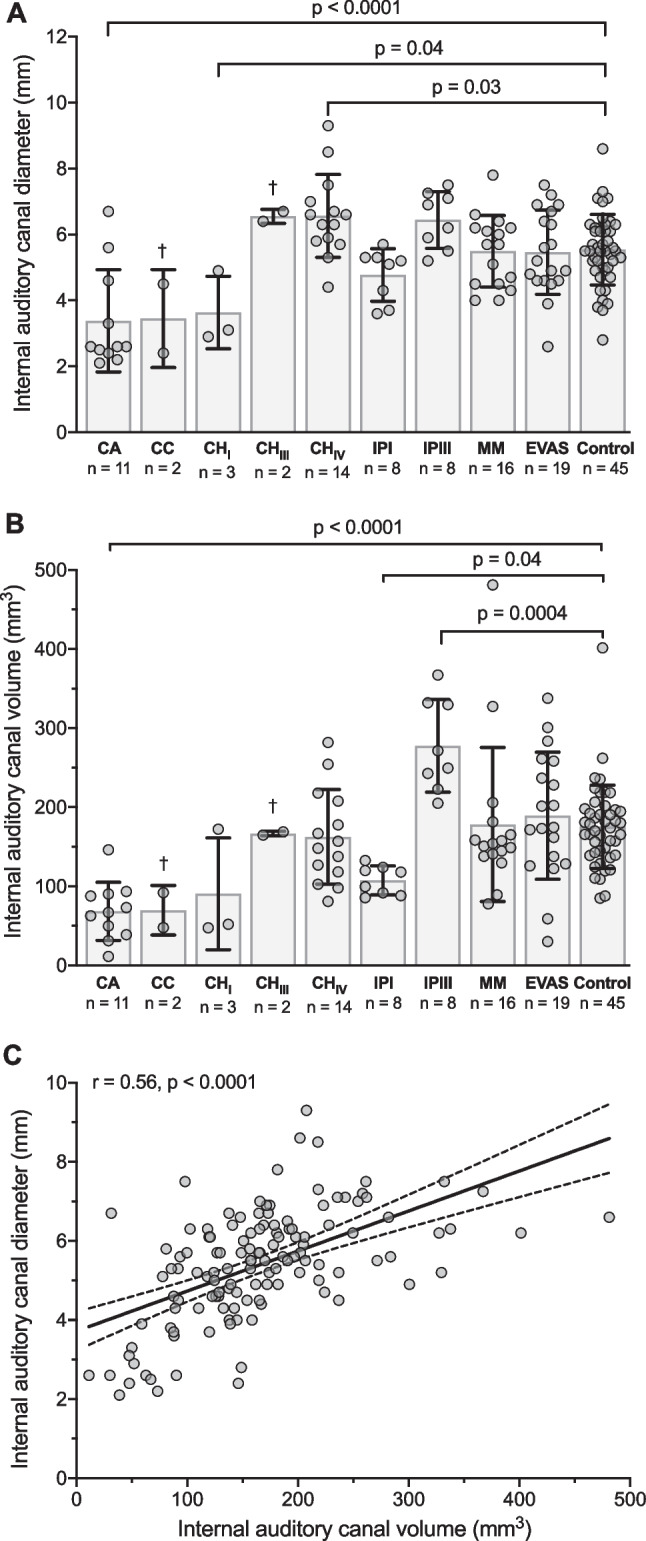
Internal auditory canal (IAC) measurements in inner ear malformations (IEMs) and controls. **A**, **B** IAC diameter (**A**) and volume (**B**) in IEMs and controls. Box indicates mean, whiskers indicate standard deviation. **C** Correlation between IAC diameter and volume in all measurements of IEMs controls. Solid black line represents linear regression line, dashed lines represent 95% prediction intervals. *r*, Pearson's correlation coefficient. *CH* cochlear hypoplasia, *IP* incomplete partition, *MM* Mondini malformation, *EVAS* enlarged vestibular aqueduct syndrome

The mean IAC diameter in controls was 5.5 mm (SD 1.1 mm). A one-way ANOVA revealed significant differences in the IAC diameter among different types of IEMs and controls (*F*(7,116) = 9.24, *p* < 0.0001; Fig. 2A). In comparison with the control group, post-hoc analysis revealed statistically significant difference in IAC diameter in CA (mean difference 2.2 mm, *p* < 0.0001), CHI (mean difference 1.9 mm, *p* = 0.04), and CHIV (mean difference -1.0 mm, *p* = 0.03). The inter-observer agreement for IAC diameter was good with an ICC of 0.77.

### Internal auditory canal volume

A three-dimensional model of the bony labyrinth and IAC of the inner ear was successfully reconstructed in every case. The mean IAC volume in controls was 175.3 mm^3^ (SD 52.6 mm^3^). A one-way ANOVA revealed significant differences in the IAC volume among different types of IEMs and controls (*F*(7,116) = 9.23, *p* < 0.0001; Fig. 2B). In comparison with the control group, post-hoc analysis revealed statistically significant difference in IAC volume in CA (mean difference 107.0 mm^3^, *p* < 0.0001), in IPI (mean difference 67.9 mm^3^, *p* = 0.04), and IPIII (mean difference − 102.2 mm^3^, *p* = 0.0004 mm^3^). Volumetric measurements of the IAC showed an excellent inter-rater reliability with an ICC of 0.92.

### Correlation between IAC volume and IAC diameter

The overall IAC volume exhibited a fair correlation to the IAC diameter (*r* = 0.56, *p* < 0.0001, Fig. 2C). Individual correlation coefficients were *r* = 0.7 (*p* = 0.01) for CA, *r* = 0.3 (n. s.) for CHIV, *r* = − 0.2 for IPI (n. s.), *r* = 0.2 (n. s.) for IPIII, *r* = 0.4 (n. s.) for MM, *r* = 0.6 (*p* = 0.005) for EVAS and *r* = 0.5 (*p* = 0.0004) for controls.

## Discussion

This study assessed volumes of the IAC in IEMs and controls with regular anatomy. Furthermore, the association of the IAC volume to the IAC diameter was investigated. Compared to controls, a significantly smaller IAC diameter was found in CA, CHI and CHIV and a significantly smaller IAC volume in CA, IPI and IPIII. Furthermore, the IAC diameter fairly correlated with the IAC volume with significant differences among the different types of IEM.

Although the IAC is often only qualitatively assessed [26], the IAC diameter at the internal auditory meatus represents a common measure to quantify the IAC size. Based on our data, we can estimate a normal IAC diameter at the level of the internal auditory meatus between 3.3 and 7.7 mm (based on the mean ± two standard deviations). This is in accordance to previous studies, which reported a mean IAC diameter between 5 and 6 mm and an IAC diameter range between 2 and 8 mm to be normal [15, 17, 29, 30]. In contrast, only few previous studies reported volumetric data on a normal IAC [31]. Furthermore, information on volumes of hypoplastic IAC as well as IAC size in IEM is unknown. Nevertheless, IAC diameter and volume of controls obtained in the present study were comparable to those reported in literature [31, 32]. In some previous studies, the IAC volume has been estimated by a formula for a cone after assessing 2D measurements [32]. However, especially in cases of IPIII, where the IAC is confluent with the cochlear base, these estimations might be difficult to apply.

Notably, the IAC volume showed a markedly higher inter-rater reliability compared to the IAC diameter. Therefore, the IAC volume may constitute the more robust measure. Interstingly, our data indicate that these two measures do not exhibit a strong correlation. Furthermore, since the IAC exhibits a highly variable shape, the IAC diameter at the internal auditory meatus may not be a suitable surrogate for the entire anatomic structure of the IAC, even in normal inner ears [33]. This is corroborated in our study by the group of IPIII cases, in which the IAC has been reported to be enlarged [34]. Whereas we did not find a significant difference in IAC diameter, the IAC volume was significantly larger in IPIII than in controls. Moreover, in IPI, we found a significantly smaller IAC volume, but a similar IAC diameter compared to controls. Although there are conflicting reports on IAC size in IPI, our results on the IAC volume seem to support the notion of a rather narrow IAC in IPI [17]. Furthermore, the IAC diameter showed statistically larger values in CHIV compared to controls, whereas this effect was not apparent in IAC volumes. Since an enlarged IAC is not described in cases of CH, volumetry may represent the more accurate measurement method than IAC diameter.

The IAC is formed by embryonic mesoderm, which ensheaths the facial and vestibulocochlear nerve and eventually undergoes ossification [35]. It is hypothesized that a hypoplastic or aplastic IAC is secondary to disrupted development of the cochleovestibular nerve [5, 35]. Therefore, the IAC morphology including size and volume may be used as a radiologic surrogate marker for the integrity of the vestibulocochlear nerve. However, it is important to note that vice versa a normal IAC does not exclude hypoplasia or aplasia of the vestibulocochlear nerve [36]. IAC size may still be a useful indicator for both cochlear nerve anomalies as well as further (inner) ear malformation, which may guide further therapy in case of sensorineural hearing loss. Although the exact relationship of a small volume of the IAC and cochlear nerve hypoplasia has yet to be established, we suggest an IAC with a volume below 70 mm^3^ as small based on the present data. Nevertheless, a small IAC should be interpreted in conjunction with the results of both additional imaging studies using MRI to visualize the IAC and cochlear nerve as well as audiometric testing [11]. This stud﻿y is limited by the fact that the morphological and functional integrity of the vestibulocochlear nerve was not assessed.

In conclusion, volumetric measurement of IAC in cases of IEMs reduces measurement variability and may add to the most widely accepted classification systems of IEM. Since a hypoplastic IAC can be associated with a hypoplastic cochlear nerve and sensorineural hearing loss, radiologic assessment of the IAC is crucial in the work-up of patients with severe sensorineural hearing loss undergoing cochlear implantation.
